# ResolvinD_1_ stimulates epithelial wound repair and inhibits TGF-*β*-induced EMT whilst reducing fibroproliferation and collagen production

**DOI:** 10.1038/labinvest.2017.114

**Published:** 2017-10-30

**Authors:** Shengxing Zheng, Qian Wang, Vijay D'Souza, Dom Bartis, Rachel Dancer, Dhruv Parekh, Fang Gao, Qingquan Lian, Shengwei Jin, David R Thickett

**Affiliations:** 1 Department of Anesthesia and Critical Care, The Second Affiliated Hospital and Yuying Children’s Hospital of Wenzhou Medical University, Zhejiang, China; 2 Institute of Inflammation and Aging, University of Birmingham, Edgbaston Birmingham, Birmingham, UK

## Abstract

Acute and chronic inflammatory lung diseases are often associated with epithelial cell injury/loss and fibroproliferative responses. ResolvinD_1_ (RvD1) is biosynthesized during the resolution phase of inflammatory response and exerts potent anti-inflammatory and promotes resolution of inflammatory lung diseases. The aim of this study was to investigate whether RvD1 exerts protective effects on alveolar epithelial cell function/differentiation and protects against fibroproliferative stimuli. Primary human alveolar type II cells were used to model the effects of RvD1 *in vitro* upon wound repair, proliferation, apoptosis, transdifferentiation, and epithelial–mesenchymal transition (EMT). Effects of RvD1 upon primary human lung fibroblast proliferation, collagen production, and myofibroblast differentiation were also examined. RvD1 promoted alveolar type II (ATII) cell wound repair and proliferation. RvD1 protected ATII cells against sFas-ligand/TNF-*α*-induced apoptosis and inhibition on cell proliferation and viability. RvD1 promoted ATII cells transdifferentiation. Moreover, we demonstrate that RvD1 inhibited EMT in response to TGF-*β*. Furthermore RvD1 inhibited human lung fibroblast proliferation, collagen production, and myofibroblast differentiation induced by both TGF-*β* and bronchoalveolar lavage fluid from acute respiratory distress syndrome (ARDS) patients. The effects of RvD1 were PI3-kinase dependent and mediated via the resolvin receptor. RvD1 seems to promote alveolar epithelial repair by stimulating ATII cells wound repair, proliferation, reducing apoptosis, and inhibiting TGF-*β*-induced EMT. While RvD1 reduced fibroproliferation, collagen production, and myofibroblast differentiation. Together, these results suggest a potential new therapeutic strategy for preventing and treating chronic diseases (such as idiopathic pulmonary fibrosis) as well as the fibroproliferative phase of ARDS by targeting RvD1 actions that emphasizes natural resolution signaling pathways.

Chronic lung diseases such as idiopathic pulmonary fibrosis (IPF) are associated with loss of alveolar epithelial cells due to apoptosis, excessive fibroproliferation, aberrant deposition of extracellular matrix (EMC), inflammation, and dysregulated repair of lung tissue.^1, 2^ Many Studies suggest alveolar epithelial cells have a central role in the pathogenesis of IPF.^3, 4, 5, 6^ In IPF, alveolar epithelial cells may undergo increased apoptosis through a Fas-ligand–mediated pathway.^7^ Injury to the alveolar epithelial barrier is an early event during the development of pulmonary fibrosis.^6^ Timely repair of lung injury is essential for proper restoration of function. The repair of a damaged alveolar epithelial barrier is a complex and poorly understood process that includes transdifferentiation of type II epithelial cells into type I epithelial cells, as well as regeneration of epithelial cells from stem cells.^8^ Dysregulation of repair mechanisms such as epithelial to mesenchymal transition, may contribute to the generation of numerous ECM-producing fibroblasts/myofibroblasts,^9, 10^ that result in clinically significant pulmonary fibrosis. Similarly, in acute lung diseases such as acute respiratory distress syndrome (ARDS), the degree of the epithelial injury and the loss of alveolar epithelial cells due to Fas-ligand–mediated apoptosis is an important predictor of outcome.^11, 12, 13^ In some cases of ARDS, a marked fibroproliferative response is associated with bad outcome.^14^ Therefore, a therapy that promotes epithelial repair and inhibits epithelial–mesenchymal transition (EMT) could be useful in both acute and chronic respiratory disease, but only if it was not also a stimulus for fibro-poliferation.

ResolvinD_1_ (RvD1) is a lipid mediator derived from both eicosapentaenoic acid and docosahexaenoic acid which acts to dampen excessive PMN infiltration and transmigration.^15^ Previous studies have suggested that RvD1 attenuates lung inflammation and can maintain the integrity of lung epithelium.^16^ Furthermore, RvD1 has recently been shown to reduce interstitial fibrosis,^17^ inhibit cytokines release at sites of inflammation,^18, 19^ and is protective after ischemia-reperfusion second organ injury.^17, 20^ RvD1 directly activates the lipoxinA_4_ receptor/formyl peptide receptor 2 (ALX/FPR2) with high affinity.^21^

Currently what is not known is whether RvD1 has a direct role in modulating human lung epithelial cell or primary human lung fibroblast proliferation and function.

Our results indicate that RvD1 promotes epithelial wound repair and inhibited TGF-*β* induced EMT in human adult type II alveolar epithelial cells, whilst inhibiting fibroproliferation and reducing the effects of TGF-*β* on primary human lung fibroblast (HLF) collagen production and myofibroblast differentiation.

## Materials and methods

### Reagents

RvD1 was purchased from Cayman chemicals (Cayman Chemical Company, USA). Recombinant human TGF-*β* was purchased from R&D (R&D Sytems, Abingdon, UK). Antibody against caspase-8, AKT and phospho-AKT were obtained from Cell Signal Technology (Cell signal Technology, Boston, USA). Antibody against E-cadherin, N-cadherin, and *α*-SMA were obtained from Abcam (Abcam, Cambridge, UK). Antibody against *β*-actin was purchased from Santa Cruz Biotechnology Inc (Santa Cruz, CA, USA).

### Primary Lung Cell Culture

Alveolar type II (ATII) cells were isolated from peripheral normal lung tissue distal from the tumor in patients undergoing lung cancer resection. The cells were isolated in accordance with approval from the local research ethics committees at the University of Birmingham (Birmingham, UK). Primary human ATII cells were extracted according to methods described previously (see online Supplementary Information).^22^ Average yields of primary human ATII cells were 30.2 million cells per resection with an average purity of 92% ATII-like cells. Cells were tested for primary human ATII cell phenotype by alkaline phosphatase staining, lysotracker lamellar body staining and by PCR expression of surfactant protein C—a type II cell marker with negative expression of aquaporin V (a type I cell marker) (data not shown).

Primary human lung fibroblasts (HLF) from Lonza were similarly cultured in Dulbecco modified Eagle medium culture media (ECACC, Sigma, Poole, UK) supplemented with 10% FCS (Sigma, Poole, UK) at 37 °C and 5% CO_2_. Cells were subcultured at 60–80% confluence using trypsin/EDTA. Cells were obtained from three separate donors, and all experiments were repeated in triplicate.

#### Stimuli and inhibitors

ATII cells and fibroblasts were treated with RvD1 (10, 25, or 100 nM; Cayman Chemical Company, USA). Appropriate vehicle controls were used for all experiments with inhibitors. Inhibitors were used at the following concentrations according to manufacturers’ instructions: LY294002, a PI3-kinase inhibitor (Calbiochem, Nottingham, UK) at 10 *μ*M; and the ALXR antagonist, Boc-2 (N-t-Boc-Phe-Leu-Phe-Leu-Phe; GenScript USA Inc), at 10 *μ*M. Inhibitors were added to cells 1 h before every treatment.

### BALF Collection

Bronchoalveolar lavage fluid (BALF) from ARDS patients is known to stimulate epithelial repair in the scratch wound assay in an IL-1*β*-dependent fashion.^23^ To test whether RvD1 could augment or synergize with this effect, the BALF from patients with ARDS were mixed 50:50 with appropriate culture media for each cell type as a positive control stimulus. We used BALF from patients enrolled into the BALTI-1 trial, demographics for whom have been published previously.^24^

### *In Vitro* Alveolar Epithelial Wound Repair Assay

Epithelial repair was determined using an *in vitro* epithelial wound repair assay as described before.^25^ Briefly, primary human ATII cells were grown to confluent monolayers before wounding with a 1-ml pipette tip. Digital images of the same point on the wound were taken at time 0 and at time 36 h. To control for the inconsistencies in wound size, only monolayers in which the original wound areas varied by 10% of the mean were analyzed. Repair is expressed as the percentage of the original wound area covered by cells relative to control media. To allow for variability between cell types and batches, data are expressed as the mean (s.e.) percentage of control).

### BRDU Cell Proliferation Assay and Cell Viability Assays

BrdU incorporation was assessed according to manufacturers’ instructions (BRDU Cell Proliferation Assay, Promega, UK). Cell Viability after 24 h was assessed adding 20 *μ*l of Cell Titer 96 aqueous one solution cell proliferation solution (Promega, UK) to cells for 1.5 h at 37 °C and 5% CO_2_ as described.^26^

### Flow Cytometry

Apoptosis of epithelial cells was assessed as described previously using flow cytometry.^22^ Cells were left in serum-free media for 24 h before exposure to 100 ng/ml Fas-ligand (R&D Sytems, Abingdon, UK). Apoptosis was determined by flow cytometry using the Annexin V and SyTOX antibody according to the manufacturer’s recommendations (Molecular Probes, Eugene, OR, USA) after 24 h exposure.

### Quantitative PCR

Quantitative PCR was performed using commercially obtained primers as outlined in Supplementary Methods.^27^

### Western Blot Analysis

Western blot analyses from cells homogenates were performed as described previously. After equal amounts of protein were electrophoresed on 10/12% SDS-PAGE and then transferred to polyvinylidene difloride membranes (Millipore, Billerica MA01821). Western blot analysis was performed using the Image Quant LAS 4000 mini (GE).

### HLF Proliferation Assay in Response to ARDS BALF

HLF were plated out at 2500 cells per well. A standard curve of cell counts was pipetted from 1250 to 15 000 per well. BALF mixed 50:50 with media was added from 10 ARDS patients. After 24 h BRDU incorporation was assessed according to manufacturers’ instructions (BRDU Cell Proliferation Assay, Promega, UK). Results were extrapolated from the standard curve. Each patient sample was run with 6 replicates upon a single batch of HLF at passage 3.

### Statistical Analysis

Data were normally distributed and analyzed by analysis of variance with Tukey’s test for *post hoc* comparisons using Minitab 14.0 (Minitab, State College, PA, USA). A *P*-value equal or less than 0.05 was considered significant. Data are expressed as mean (s.e.m.).

## Results

### RvD1 Stimulates ATII Cell Wound Repair and Proliferation *In Vitro*

**Figure 1:**
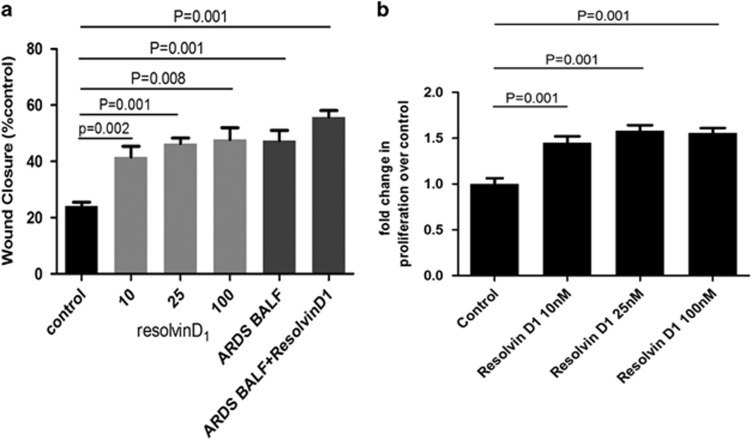
RvD1 stimulates ATII cells wound repair and proliferation *in vitro*. (**a**) RvD1 at different concentrations was added to monolayers of ATII cells physically wounded with a 1-ml pipette tip. To allow for variability between cell batches, data are expressed as the mean (s.e.) percentage of the baseline wound size for each separate set of experiments for each culture condition. (**b**) RvD1 stimulated the proliferation of primary human ATII cell. Values of >1 fold of control reflect increased proliferation. *N*=6 for each culture condition, repeated using cells from three donors.

RvD1 increased ATII cell wound closure after 36 h compared with control media. ARDS BALF increased ATII cell wound closure compared with media control.^23^ RvD1+ARDS BALF further enhanced the wound repair response (Figure 1a).

Scratch wound repair can occur due to either spreading of cells and/or proliferation. Cell proliferation studies confirmed that RvD1 stimulated proliferation of ATII cell in a dose-dependent manner (Figure 1b).

### RvD1 Promotes ATII Cell Proliferation Through Activation of ALX Receptor and the PI3K/AKT Signaling Pathway

**Figure 2:**
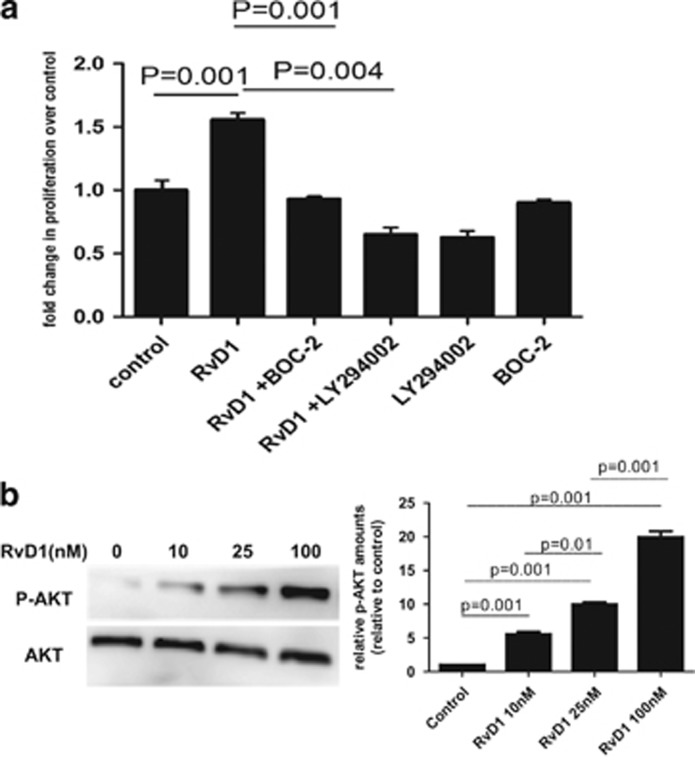
RvD1 promotes ATII cells proliferation through activation of ALX receptor and the PI3K/AKT signaling pathway. (**a**) 100 nM RvD1 promoted proliferation of ATII cells. Pre-treatment with 10 *μ*M LY294002, a phosphatidylinositol 3′-kinase/Akt inhibitor inhibited the effects of RvD1 on ATII cells proliferation of suggesting that the pro-proliferation effects of RvD1 are PI3-kinase dependent. BOC-2, the ALX receptor antagonist, was re-incubated with primary human ATII cells at 10 *μ*M for 1 h before RvD1 treatment of ATII cells. BOC-2 treatment inhibited the effects of RvD1 on the proliferation of primary human ATII cells suggesting that the promoting proliferation effects of RvD1 are ALX receptor dependent. (**b**) To investigate whether RvD1 can activate AKT phosphorylation in ATII cells, ATII cells were stimulated with different concentrations of RvD1 (10, 25, and 100 nmol/ml) for 24 h. We found that RvD1 activated AKT phosphorylation in a dose-dependent manner.

The PI3K/AKT signaling pathway has an important role in cell proliferation. To determine if PI3-kinase signaling was involved in the RvD1 proliferative response, LY294002 (PI3K inhibitor) was incubated with ATII cells at 10 *μ*M for 1 h before RvD1 treatment of ATII cells. LY294002 treatment reversed the effects of RvD1 (100 nM) on the proliferation of ATII cells compared with control media-treated cells (Figure 2a). To investigate whether RvD1 can activate AKT phosphorylation in ATII cells, ATII cells were stimulated with different concentrations of RvD1 (10, 25, and 100 nmol/ml) for 24 h. We found that RvD1 activated AKT phosphorylation (Figure 2b). In addition, Pre-treatment of cells with Boc-2 (the resolvin ALX receptor antagonist) inhibited the effects of RvD1 on the proliferation of ATII cells (Figure 2a).

### RvD1 Protects Against Fas-ligand and TNF-*α* Actions on ATII Cells

**Figure 3:**
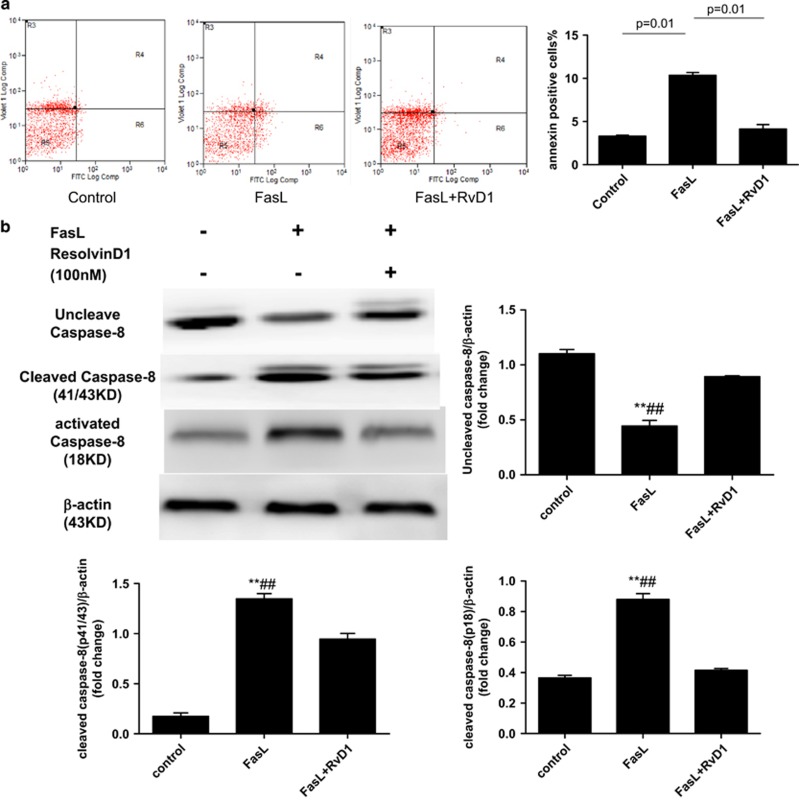
Effect of RvD1 upon effects of soluble Fas-ligand and TNF-alpha on apoptosis and caspase-8 activation. (**a**) Flow cytometry analysis of annexin-positive cells 24 h after treatment with 100 ng/ml sFasL. Co-treatment with RvD1 at 100 nM reduced annexin binding. sFasL treatment of ATII cells increased the number of apoptotic cells from 3.29±0.11% in control cells to 10.34±0.33% (*P*=0.01). Rescue treatment with RvD1 reduced the number of apoptotic cells to 4.12±0.52% (*P*=0.01). (**b**) RvD1 inhibited sFasL-induced caspase-8 activation on ATII cells. Western blots showing caspase-8 protein in ATII cells treated with 100 ng/ml sFasL or/and RvD1 at 100 nM for 24 h. Caspase-8 protein was quantified and analyzed in the indicated groups. **P<0.01 relative to control group, ^##^*P*<0.01 compared with FasL group.

Soluble Fas-ligand (sFasL) and TNF-*α* inhibited cellular proliferation compared with control media-treated cells. This effect was attenuated by 100 nM RvD1 pre-treatment (see online Supplementary Figures 1A). The addition of 100 ng/ml sFasL or 100 ng/ml TNF-*α*, as expected, significantly reduced cellular viability compared with control group. Co-treatment with 100 nM RvD1 significantly increased cellular viability compared with sFasL (*P*<0.01; see online Supplementary Figures 1B) an effect that was also reduced by RvD1 rescue therapy added 30 min after treatment with sFasL (data not shown). sFasL treatment of ATII cells (which are known to be resistant to apoptosis) increased the number of apoptotic cells from 3.29±0.11% in control cells to 10.34±0.33% (*P*=0.01). Rescue treatment with RvD1 reduced the number of apoptotic cells to 4.12±0.52% (*P*=0.01; Figure 3a).

### RvD1 Inhibited Fas-ligand-induced Caspase-8 Activation in ATII Cells

To research the impact of RvD1 on Fas-ligand induced ATII cells apoptosis, we preliminarily detected the caspase-8 levels *in vitro*. ATII cells were treated with Fas-ligand or/and RvD1. The protein levels of caspase-8 were quantified and analyzed in the indicated groups (Figure 3b). Fas-ligand promoted caspase-8 activation. RvD1 inhibited Fas-ligand induced caspase-8 activation in ATII cells.

### RvD1 Promotes Aquaporin 5 Gene Expression Whilst Inhibiting Surfactant Protein C Gene Expression on ATII Cells

**Figure 4:**
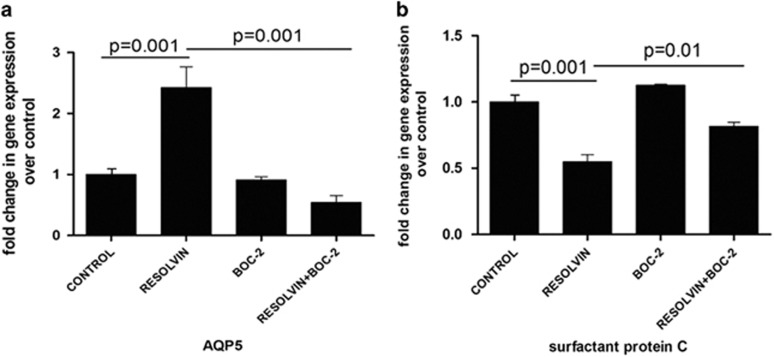
RvD1 upregulates Aquaporin V and downregulates SP-C. To observe the effect of RvD1 on AQP5 and surfactant protein C gene expression, ATII cells were treated by RvD1 for 24 h. (**a**) Aquaporin 5 gene expression: RvD1 only (2.42±0.34 fold) relative to control group, *P*=0.001. The effect that can be blocked by BOC-2 (the FPR antagonist; resolvinD1+BOC-2, mean, 0.54±0.11 fold, *P*=0.001). (**b**) Surfactant ptotein C gene expression: RvD1 only (0.54±0.05-fold) relative to control group, *P*=0.001. The effect that can be blocked by BOC-2 (the FPR antagonist) (RvD1+BOC-2, mean, 0.82±0.03 fold, *P*=0.01).

Aquaporin 5 (AQP5, a type I epithelial cell marker) is a membrane protein that mainly facilitates osmotic water transport.^28^ AQP5 may promote alveolar fluid clearance or maintain integrity of epithelial barrier.^29^ Roles of AQP5 other than fluid transport have been explored in animal lung injury models, results from these studies show that lung injury is associated with down-regulation of AQP5 expression.^30, 31^ AQP5 expression has been used as a type I epithelial cell marker. To observe the effect of RvD1 on AQP5 and Surfactant Protein C (SP-C, a type II epithelial cell marker) gene expression, ATII cells were treated by RvD1 for 24 h. RvD1 increased AQP5 gene expression (2.42±0.34 fold) relative to control group, *P*=0.001 (Figure 4a). RvD1 induced AQP5 expression was blocked by BOC-2 (the ALXR antagonist; Figure 4a). In contrast, RvD1 downregulated mRNA expression of SP-C suggesting that RvD1 may promote transdifferentiation of ATII cells into ATI like cells (Figure 4b).

### RvD1 Inhibit TGF- *β* -Induced EMT in PRIMARY Human Alveolar Type II Cells

**Figure 5:**
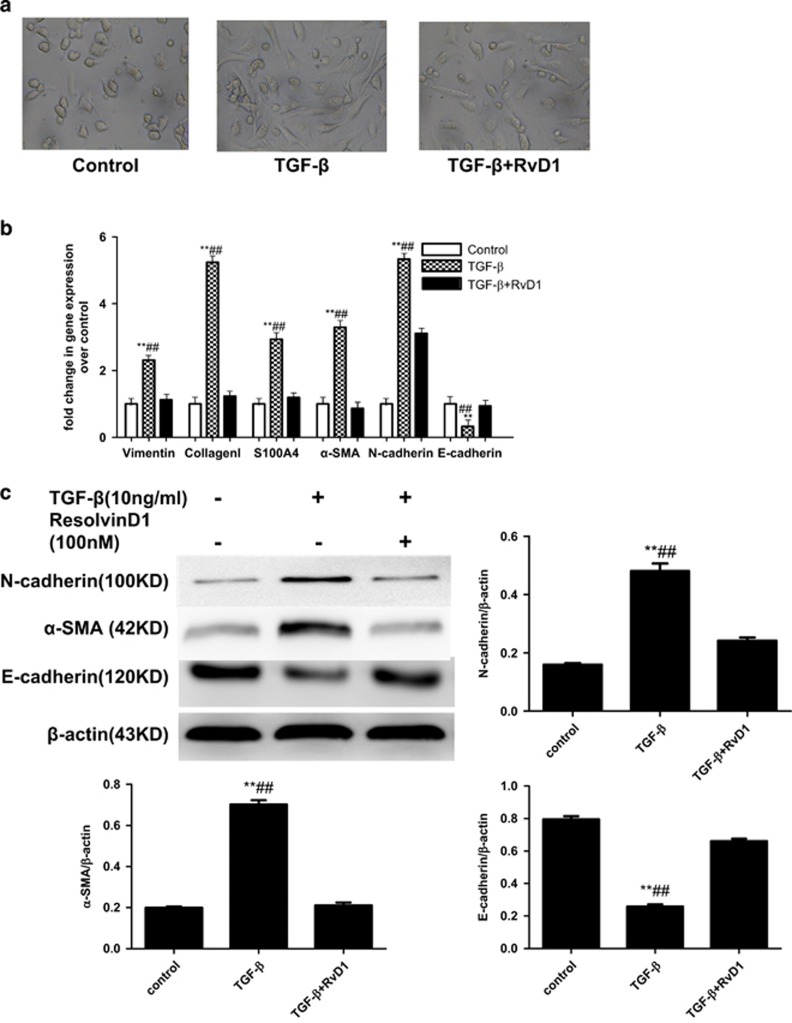
RvD1 inhibits TGF-*β-*induced EMT in Primary human alveolar type II cells. (**a**) TGF-*β-*treated ATII cells showed a mesenchymal morphology (fibroblast-like), and RvD1 restored the epithelial morphology of the cells to a certain extent. (**b**) ATII cells were pre-treated with RvD1 for 2 h. The cells were then cultured with TGF-*β* (10 ng/ml) for 48 h. *β*-Actin was used here as an internal control. EMT was induced with TGF-*β* treatment. TGF-*β* treatment induced the expression of mRNA of mesenchymal markers including N-cadherin, vimentin, type I collagen, S100A4, and *α*-SMA, and reduced the expression of epithelial markers such as E-cadherin. RvD1 blocked the expression of mRNA of mesenchymal markers including N-cadherin, vimentin, type I collagen, S100A4, and *α*-SMA, while RvD1 restored the expression of mRNA of E-cadherin, **P<0.01 relative to control group respectively, ^##^*P*<0.01 compared with TGF-*β* group respectively. (**c**) The effects of RvD1 on the TGF-*β*-treated E-cadherin, *α*-SMA, N-cadherin of ATII cells were confirmed by western blot. **P<0.01 relative to control group respectively, ^##^*P*<0.01 compared with TGF-*β*+ RvD1 group respectively.

The EMT of alveolar epithelial cells is a phenotype conversion, which is one of the main mechanisms of pulmonary firosis.^32^ The EMT process of epithelial cells is often stimulated with TGF-*β*.^32^ In our study, EMT was induced in ATII cells with TGF-*β* treatment. TGF-*β* treated ATII cells showed a mesenchymal morphology (fibroblast-like), and RvD1 restored the epithelial morphology of the cells to a certain extent (Figure 5a). RvD1 blocked the expression of mRNA of mesenchymal markers including N-cadherin, vimentin, type I collagen, S100A4, and *α*-SMA, while RvD1 restored the expression of mRNA of E-cadherin (Figure 5b). The effects of RvD1 on the TGF-*β*-treated E-cadherin, *α*-SMA, N-cadherin of ATII cells were confirmed by western blot (Figure 5c).

**Figure 6:**
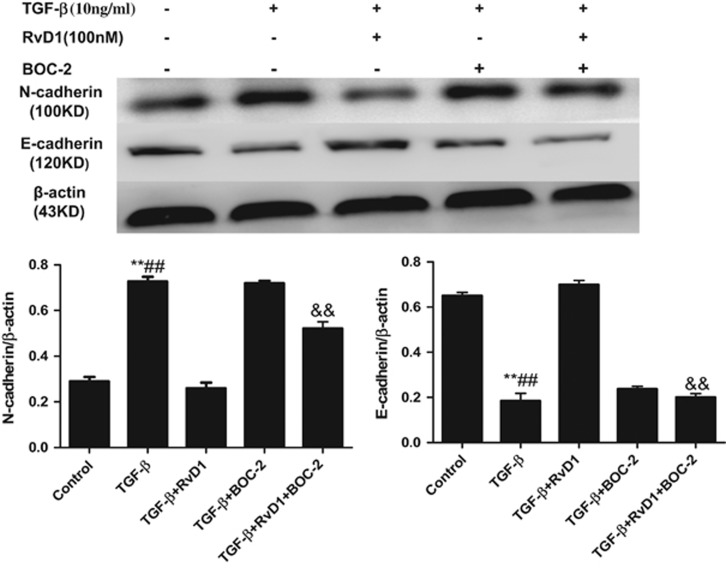
RvD1 inhibits TGF-*β* induced EMT in primary human alveolar type II cells via ALX/FPR2 receptor. To elucidate the mechanism involved in the effects of RvD1 on EMT, pre-treatment of cells with Boc-2 (the ALX receptor antagonist), inhibited the effects of RvD1 on EMT of ATII cells. TGF-*β*-induced N-cadherin expression was suppressed by RvD1, and pre-treatment of cells with Boc-2 incapacitated the effects of RvD1 on N-cadherin expression. Reduced expression of E-cadherin in TGF-*β*-treated ATII cells was restored by RvD1, but pre-treatment of cells with Boc-2 abolished the effects of RvD1. **P<0.01 relative to control group respectively, ^##^*P*<0.01 compared with TGF-*β*+RvD1 group respectively, ^&&^*P*<0.01 compared with TGF-*β*+RvD1 group respectively.

To elucidate the mechanism involved in the effects of RvD1 on EMT, pre-treatment of cells with Boc-2 (the ALX receptor antagonist), inhibited the effects of RvD1 on EMT of ATII cells. TGF-*β*-induced N-cadherin expression was suppressed by RvD1, and pre-treatment of cells with Boc-2 incapacitated the effects of RvD1 on N-cadherin expression (Figure 6). Reduced expression of E-cadherin in TGF-*β*-treated ATII cells was restored by RvD1, but pre-treatment of cells with Boc-2 abolished the effects of RvD1 (Figure 6).

### RvD1 Inhibits Proliferation of PRIMARY HLF Induced by TGF- *β* and this Effect was PI3-Kinase Dependent and Blocked by BOC-2

**Figure 7:**
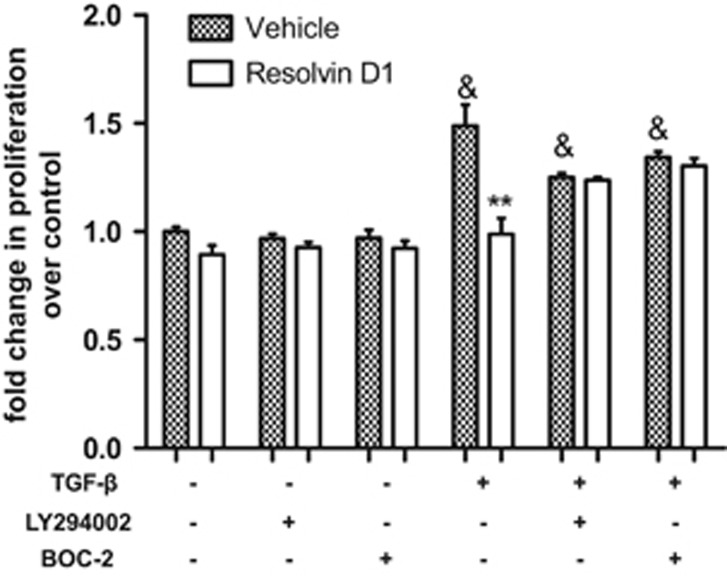
Effect of RvD1 on primary HLF proliferation in response to TGF-*β*. Cell proliferation studies confirmed that RvD1 inhibited proliferation of primary HLF induced by TGF-*β*. Cultured and serum-deprived cells were treated with 10 ng/ml TGF-*β* for 24 h with or without pre-incubation with LY294002 (10 *μ*M) for 1 h, BOC-2 (10 *μ*M) for 1 h. Data are mean±s.e.m. of three independent experiments. ^&^<0.05, compared with no treatment group ; **P<0.01, compared with TGF-*β* only group.

Cell proliferation studies confirmed that 100 nM RvD1 inhibited proliferation of primary HLF Induced by TGF-*β*. Cells were treated with TGF-*β* for 24 h with or without pre-incubation with the PI3- kinase inhibitor LY294002 (10 *μ*M) or BOC-2 (10 *μ*M). RvD1 inhibited the effects of TGF-*β* on HLF proliferation, and these effects were blocked by both LY294002 and BOC-2 (Figure 7).

### RvD1 Inhibits Proliferation of Primary HLF-Induced BALF from Patients with ARDS

**Figure 8:**
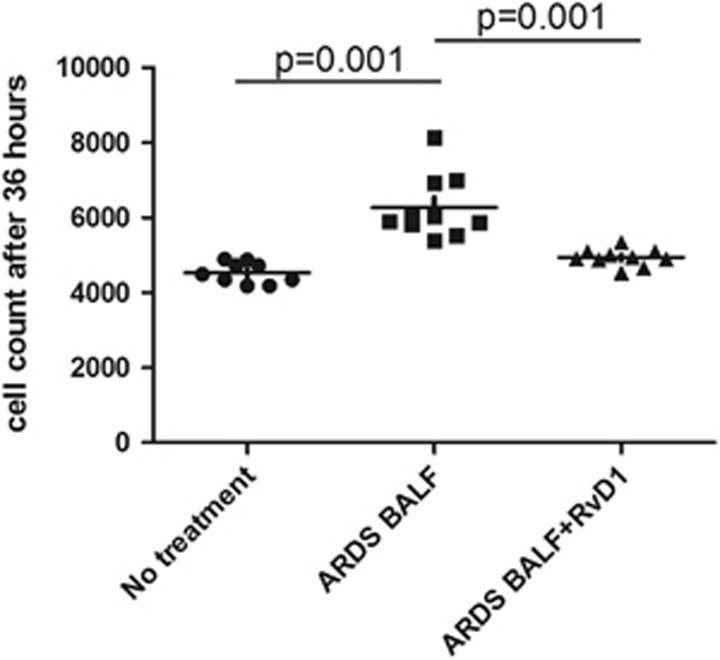
Effect of RvD1 on primary HLF proliferation in response to ARDS BALF. BALF from patients with ARDS stimulated proliferation of primary HLF. RvD1 inhibited the proliferation of primary HLF-induced BALF from patients with ARDS. Data are mean±s.e.m. of three independent experiments.

ARDS BALF has previously been shown to promote fibroblast proliferation *in vitro*.^33^ To model the *in vivo* stimulus for fibroproliferation in ARDS, HLF were treated with a 50:50 mix of BALF from patients with ARDS. RvD1 inhibited ARDS BALF induced proliferation in HLF (Figure 8).

### RvD1 Reduces Primary HLF Collagen Production and *α*-SMA Induced by TGF- *β* and BALF from Patients with ARDS

We investigated the mRNA expression of type I collagen, type IV collagen, and *α*-SMA in HLF induced by TGF-*β* 10 ng/ml with quantitative PCR. Gene expression of type I collagen, type IV collagen, and *α*-SMA were increased in HLF induced by 24 h of treatment with TGF-*β* relative to control group. Treatment with RvD1 significantly inhibited gene expression of type I collagen, type IV collagen, and *α*-SMA in HLF induced by TGF-*β* compared with TGF-*β* group, respectively (Table 1). We also investigated the effect of ARDS BALF upon type I collagen, type IV collagen, and *α*-SMA mRNA expression. Gene expression of type I collagen, type IV collagen, and *α*-SMA were increased in HLF induced by ARDS BALF relative to control group (Table 1). RvD1 significantly inhibited gene expression of type I collagen, type IV collagen, and *α*-SMA in HLF induced by ARDS BALF (Table 1).

## Discussion

Epithelial injury is one of the hallmarks of ARDS and fibrotic lung diseases.^1, 34^ Progressive pulmonary fibrosis occurs due to recurrent injury to AECs followed by aberrant repair/regeneration of epithelial barrier, persistence of activated fibroblasts, and alterations in ECM.^1, 35^ It has been suggested that RvD1 exerts potent anti-inflammatory and proresolution effects, without causing immunosuppression. Furthermore, we previously reported that RvD1 improved alveolar fluid clearance, decreased pulmonary edema and maintained the integrity of lung epithelia in LPS-induced murine lung injury.^36^ Aspirin-triggered RvD1 also improved epithelial and endothelial barrier integrity in a murine model of hydrochloric acid-induced ALI.^37^ RvD1 promoted macrophage phagocytosis of zymosan and apoptotic PMNs signaling through the human ALX and GPR32 receptors.^21^ Based on this background, our purpose was to evaluate whether RvD1 stimulated physical wound repair, promoted cellular proliferation, and inhibited apoptosis in ATII cells processes that are dysregulated repair in ARDS and fibrotic lung diseases.

The restoration of the alveolar epithelial barrier is a critical aspect of alveolar repair,^25^ and our study clearly demonstrated that RvD1 stimulated alveolar repair promoting physical wound closure by inducing proliferation of primary alveolar epithelial cells. The PI3K-AKT signaling pathway regulates proliferation. Recombinant mouse osteopontin induces the proliferation of human bronchial smooth muscle cells via the PI3K/AKT signaling pathway. ^38^ Our study also showed that the mitogenic response of ATII cells to RvD1 is mediated through activation of ALX receptor and the PI3K/AKT signaling pathway.

However, the precise role of RvD1 as modulators of apoptosis remains elusive and it is also unclear whether the effects relate to increasing lung epithelial survival. Previous studies reported that RvD1 can stimulate apoptosis of T cells and PMNs, while our study demonstrated that RvD1 inhibited apoptosis in sFasL-treated cells. One study has also indicated that RvD1 decreased apoptosis induced by ER stress in HepG2 cells.^39^ Our study demonstrates that RvD1 reduced cell death in response to sFasL and/or TNF-*α* even when given after the onset of injury and, therefore, may have potential as a rescue therapy post-injury. Furthermore, these effects seemed to relate to caspase-8 activation as caspase-8 levels were elevated in the sFasL-treated cells, and RvD1 suppressed sFasL-induced caspase-8 activation in ATII cells.

The normal alveolar epithelium is composed of two types of cells (ATI and ATII). The repair of the epithelial barrier is believed to involve the transdifferentiation of type II cells into type I epithelial cells.^8^ The inability of type II AECs to transdifferentiate into type I AECs have also been observed in human lung fibrosis.^40^ Our study showed that RvD1 promoted gene expression of AQP5 (type I marker) in ATII cells via activation of ALX supporting a potential role for RvD1 in promoting fluid transport. while RvD1 reduced gene expression of surfactant protein C (type II markers) in ATII cells suggesting that it may promote transdifferentiation of ATII cells towards the ATI phenotype. Otherwise, recurrent alveolar epithelial cell (AEC) injury that leads to aberrant activation of AEC (such as EMT), producing fibroblasts and myofibroblasts.^9, 10^

EMT has been increasingly proposed as one of the causative mechanisms of lung fibrosis.^41, 42^ In this study, we demonstrated that RvD1 restored the epithelial morphology of the cells to a certain extent. Our results also revealed that RvD1 inhibited TGF-*β* induced expression of the mesenchymal markers N-cadherin, vimentin, (FSP)-1 (also called S100A4), type I collagen and a-SMA (the mesenchymal cell markers) mRNA expression, while maintaining the epithelial marker E-cadherin (the epithelial cell marker) mRNA expression in ATII cells. Effects that were confirmed by Western blot. These results suggest that RvD1 may be a suppressor of TGF-*β*-induced EMT in ATII cells. These effects were inhibited by pre-treatment of ATII cells with Boc-2 (the resolvin ALX receptor antagonist). The role of RvD1 on EMT has also been documented in A549 cells lung cancer cells.^43^

Recent studies have indicated that 17(R)-RvD1 attenuated bleomycin-induced pulmonary fibrosis by promoting the resolution of neutrophilic inflammation in mice.^44^ Treatment with 17(R)-RvD1 attenuated neutrophil alveolar infiltration, lung collagen content, and type I collagen mRNA expression, which was inhibited by an antagonist of ALX/FPR2 receptor.^44^ RvD1 can suppress renal fibrosis in the obstructed kidney via inhibiting fibroblast proliferation and production of fibronectin and collagen I.^45, 46^ We therefore studied the effects of RvD1 upon primary HLF. RvD1-inhibited TGF-*β*-induced proliferation in primary HLF via activation of ALX/FPLR-1 and this effect was mediated through the PI3/Akt signaling pathway.

It is established that TGF-*β*, as a potent inducer of fibroblast differentiation into myofibroblasts, can stimulate fibroblast proliferation and collagen production.^47, 48^ We therefore addressed the possibility that RvD1 may inhibit their differentiation into myofibroblasts. RvD1 significantly inhibited gene expression of type I collagen, type IV collagen, and *α*-SMA (a reliable myofibroblast marker) in HLF induced by TGF-*β*. We also showed ARDS BALF stimulated the expression of markers of myofibroblast differentiation when incubated with normal HLF; an effect that was blocked by RvD1. Our data therefore suggests that RvD1 has differential effects upon ATII and HLF cells *in vitro*; both promoting epithelial repair and inhibiting TGF-*β*-induced EMT whilst reducing fibroproliferation. This differential effect is potentially vitally important for RvD1 if used as a novel therapy in both acute and chronic inflammatory lung diseases such as ARDS and IPF.

**Figure 9:**
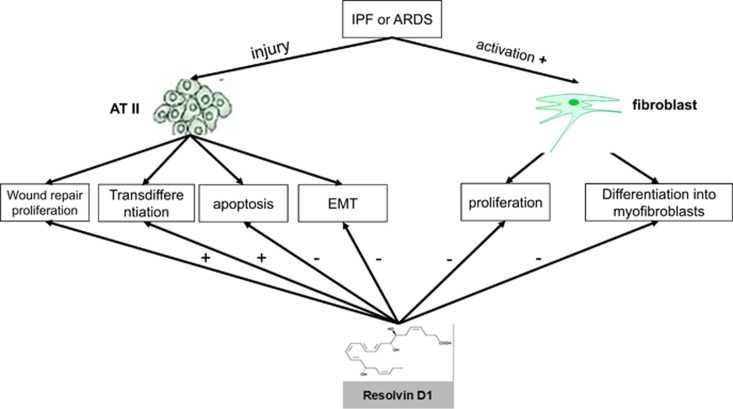
The key role for RvD1 during primary human ATII cells and HLF *in vitro*.

In summary, these data provide evidence for a new mechanism by which RvD1 may contribute to alveolar repair promoting physical wound closure by inducing proliferation of primary human ATII cells *in vitro*. RvD1 protected ATII cells from pro-apoptotic stimuli even when given after the initial injury. RvD1 increased the expression of the type I marker, AQP5, with reduction in SP-C by ATII-like cells, potentially promoting transdifferentiation. Moreover, RvD1 inhibited EMT in response to TGF-*β*. Intriguingly RvD1 also inhibited HLF proliferation, collagen production and *α*-SMA expression induced by both TGF-*β* and ARDS BALF. The potential key role for RvD1 during primary human ATII cells and HLF *in vitro* is summarized in Figure 9. Our next step is to determine whether RvD1 has a role in regulating repair and EMT in animals models.

In conclusion, these results suggest a potential new therapeutic strategy for preventing and treating chronic diseases (such as IPF) as well as the fibroproliferative phase of ARDS by targeting RvD1 actions that emphasizes natural resolution signaling pathways. Further experiments are necessary to understand the basic mechanism underlying the anti-fibrotic and anti-apoptotic effects of RvD1, which are currently under investigation.

## Figures and Tables

**Table 1:** Summary of the different types gene expression in response to treatment of different stimulations

**Gene type**	**TGF-***β*	**RvD1+TGF-***β*	**BALF**	**RvD1+BALF**
Type I collagen	323.35±168.07-fold*	12.64±1.40-fold#	9.47±3.78-fold*	8.27±2.77-fold^&^
Type IV collagen	27.04±14.46-fold*	9.94±5.77-fold#	13.48±3.67-fold*	1.95±0.99-fold^&&^
*α*-SMA	22.36±2.93-fold**	11.42±0.47-fold#	4.01±1.05-fold*	1.54±0.81-fold^&^

Abbreviations: *α*SMA, *α*-smooth muscle actin; BALF, bronchoalveolar lavage fluid; RVD1, resolvinD_1_.

Data are presented as mean±s.e.m. fold change in gene expression over control. Data are mean±s.e.m. of three independent experiments. *P<0.05 and **P<0.01 relative to control group, respectively; ^#^*P*<0.05 compared with TGF-*β* group, respectively; ^&^*P*<0.05 and ^&&^*P*<0.01 compared with the BALF group, respectively. RvD1 reduced primary HLF collagen production and *α*-SMA induced by TGF-*β* and BALF from patients with ARDS. Gene expression of type I collagen, type IV collagen and *α*-SMA were increased in HLF induced by TGF-*β* relative to control group respectively. RvD1 significantly inhibited gene expression of type I collagen, type IV collagen and *α*-SMA in HLF induced by TGF-*β* compared with TGF-*β* group respectively. Gene expression of type I collagen, type IV collagen and *α*-SMA were increased in HLF induced by ARDS BALF relative to control group respectively. RvD1 significantly inhibited gene expression of type I collagen, type IV collagen, and *α*-SMA in HLF induced by BALF from patients with ARDS (compared with BALF) group respectively.
