# Supplementary material for: ResolvinD1 stimulates epithelial wound repair and inhibits TGF-β-induced EMT whilst reducing fibroproliferation and collagen production
Source: Lab Invest. 2017 Oct 30;98(1):130–40. doi: 10.1038/labinvest.2017.114 (PMC5754464; doi:10.1038/labinvest.2017.114)
Supplement: Supplementary Information [file labinvest2017114x1.pdf]

Material and methods:

Ethics

Lung tissue was obtained as part of the Midlands Lung Tissue Collaborative. All procedures in this study were carried out in accordance with approval from the local research ethics committees at the University of Birmingham (Birmingham, UK). All patients gave written informed consent for the use of their tissue and clinical data for research purposes.

Lung resection specimens

Alveolar type II cells were extracted from patients undergoing lung cancer resection. We used cells from 14 donors for ATII cell extraction who had normal lung function (8M:6F, mean age 62.7 years). Resected lung specimens were immediately examined in the operating theatre by a member of the surgical team and a portion of specimen without macroscopic pathology and not required for a diagnostic purpose was passed to the research team. This sample was immediately immersed in sterile 0.9% saline in a sealed container and transported on ice to the laboratory for processing. The sample was measured and superficially washed with 0.9% saline immediately on arrival at the laboratory.

Primary human alveolar type II cells isolation and culture.

Primary human alveolar type II (AT II) cells were extracted according to methods described previously[22]. Briefly, Wash the tissue (lung section)

with sterile saline to remove blood and debris from the surface. Place the tissue into a fresh Petri dish ready for trypsinisation. Instill the trypsin solution (10-15 ml/5cm^3 piece, Gibco 25300) into the lung tissue in exactly the same way as the saline lavage. Place the covered Petri dish into a 37 °C incubator for 15 min. Repeat the procedure twice more to give a total trypsinisation period for 45 min. Chop the tissue finely into 1-2 mm^3 in the presence of FCS (10ml/5cm^3 piece) and DNase I (250 – µg/ml HBSS; Sigma DN25, HB9394). Shake the minced tissue suspension vigorously by hand for 5 min to enhance type II cell recovery. Filter the tissue suspension through a large gauge mesh (400-500 µm) and then a 40 µm cell filter (BD Biosciences) to remove undigested tissues and debris from the enzymatically-released epithelial cells, which pass through the filter. Centrifuge the filtrate, containing mostly single cells, at 300g at 12°C for 7 min. Suspend the cell pellets in 50-100 ml 50% DCCM-1 and 50% HBSS containing 100 µg/ml DNase I. Plate the resuspended cell suspension into either T-75 or T-175 culture flasks and incubate at 37°C for 1.30 h to enable any contaminating macrophages to adhere. Remove the media containing the nonadherent type II cell-enriched cell population and centrifuge at 300g at 12°C for 7 min. Resuspend the cell pellet in 3 ml of red cell lysis buffer and incubate for 3 min. Add enough HBSS to make up the volume and centrifuge the filtrate at 300g at 12°C for 7 min. Resuspend the cell pellet in a known small volume of (5 ml) 10% DCCM-1(Biological Industries Ltd. Kibbutz Beit-Haemek, Israel) and make up the volume 10 ml. Count the epithelial cells using as haemocytometer by

phase contrast microscopy. Filter again if clumps were found. Prepare a cytospin for alkaline phospatase staining if required. Add 10% DCCM-1 so that the cells are 1 x 10^6 epithelial cells/ml and plate onto collagen-coated plates; 1 x 10^6/well of a 6 well plate (for western blot studies), 0.5 x 10^6/well of a 24 well plate(for PCR, wound repair and Flow Cytometry Analysis) and 0.4 x 10^5/ well of a 96-well plate (make media up to 1ml/well, 500µl/well and 200 µl/well, respectively). After 24 h remove the media and nonadherent cells. Do not wash, leave remaining loosely attached cells and apply fresh 10% DCCM-1 medium. After another 16-24 h, remove medium and wash off remaining loose cells with HBSS. Apply fresh complete medium. The cells from a confluent monolayer within 3 days of plating. Average yields of primary human alveolar type II cells were 30.2 million cells per resection with an average purity of 92%. Cells were tested for primary human alveolar type II (AT II) cell phenotype by alkaline phosphatase staining, lysotracker lamellar body staining and by PCR expression of surfactant protein C—a type II cell marker with negative expression of aquaporin V (a type I cell marker) (data not shown). 0.5 Million cells were seeded onto 24 well plates and grown for 3 days in DCCM-1 (Biological Industries Ltd. Kibbutz Beit-Haemek, Israel) media supplemented with 10% fetal calf serum (FCS). Before stimulation cells were serum starved overnight (0.1% FCS) and stimulated in medium containing 0.1% FCS for 24 hours.

Primary human lung fibroblasts (HLF) were similarly cultured in dulbecco modified Eagle medium culture media(ECACC, Sigma, Poole,UK)

supplemented with 10% FCS (sigma) at 37 °C and 5% CO_2. Cells were subcultured at 60-80% confluence using trypsin/EDTA.Cells were abtained from three separate donors, and all experiments were repeated in triplicate.

Stimuli and Inhibitors

AT II cells and fibroblasts were treated with resolvinD_1 (Cayman Chemical Company,USA) at different concentrations. Inhibitors were used at the following concentrations according to manufacturers' instructions: LY294002, a PI3-kinase inhibitor (Calbiochem, Nottingham, UK) at 10 μM; and the nonselective FPR antagonist, Boc-2 (N-t-Boc-Phe-Leu-Phe-Leu-Phe; GenScript USA Inc), at 10μM. Inhibitors were added to cells 1hour prior to every treatment.

Bronchoalveolar Lavage Fluid Collection

BALF from ARDS patients is known to stimulate epithelial repair in the scratch wound assay in an IL-1 dependent fashion.[23] To test whether resolvin D1 could augment or synergise with this effect, the BALF from patients with ARDS were mixed 50:50 with appropriate culture media for each cell type as a positive control stimulus. We used BALF from patients enrolled into the BALTI-1 trial, demographics for whom have been published previously.[24]

In Vitro Alveolar Epithelial Wound Repair Assay. Epithelial repair was determined using an in vitro epithelial wound repair assay as described before.[25] Briefly, primary human alveolar type II (AT II) cells were grown to confluent monolayers before wounding with a 1-mL pipette tip. Cells were serum starved for 24 hours before wounding. After wounding, fresh basal media, bronchoalveolar lavage fluid and RvD1 at different concentrations was added to the wounded monolayers. Digital images of the same point on the wound were taken at time 0 and at time 36 hours. Images were then analyzed using the Scion Image program by an operator blinded to the treatment conditions to avoid bias. To control for the inconsistencies in wound size, only monolayers in which the original wound areas varied by 10% of the mean were analyzed. Repair is expressed as the percentage of the original wound area covered by cells relative to control media. To allow for variability between cell types and batches, data are expressed as the mean (SE) percentage of control).

BRDU cell proliferation assay

4 $X10^5$cells/ml (AT II cells) or 1.5 $X10^5$cells/ml (HLF) were seeded into a 96 well culture dish. BrdU Label was added and cells were incubated with RvD1 or TGF-β (R&D Systems). After 24 hours culture, BrdU incorporation was assessed according to manufacturers' instructions (BRDU Cell Proliferation Assay, Promega, UK).

Cell Titer Assay

After 24 hours of culture, viable cell count was assessed by adding 20µL of Cell Titer 96 AQueous One Solution Cell Proliferation solution (Promega) to cells for 1.5 hours at 37°C and 5% CO_2. Data from proliferation bioassays comparing the Cell Titer 96 Aqueous Assay and hydrogen-3–thymidine incorporation show similar results.[21] Furthermore, in preliminary experiments, there was a linear relationship between cell titer readings and manually counted trypsinized primary AT II cells/primary lung fibroblast cells over a range of cell counts (data not shown).

Western Blot Analysis: Western blot analyses from cells homogenates were performed as described previously. After equal amounts of protein were electrophoresed on 10/12% sodium dodecyl sulfate-polyacrylamide gels and then transferred to polyvinylidene difloride membranes (Millipore, Billerica MA01821). Western blot analysis was performed using the Image Quant LAS 4000 mini (GE). Antibody against caspase-8, AKT and phospho-AKT were obtained from Cell Signal Technology (Cell signal Technology, Boston, USA). Antibody against E-cadherin, N-cadherin and α -SMA were obtained from Abcam (Abcam, Cambridge, UK). Antibody against β-actin was purchased from Santa Cruz Biotechnology Inc (Santa Cruz, CA, USA).

带格式的: 默认段落字体, 字体: (默认) Verdana, (中文) +中文正文, 小四, 字体颜色: 自动设置, 图案: 清除

带格式的: 字体: Verdana, 小四, 字体颜色: 自动设置

带格式的: 字体: (默认) Verdana, (中文) +中文正文, 小四, 字体颜色: 自动设置, 图案: 清除

带格式的: 字体: Verdana, 小四, 字体颜色: 自动设置

删除的内容: .

Real time PCR Assay

Real time PCR was performed using total RNA from primary human alveolar type II (AT II) cells and fibroblats (RNeasy Mini Kit; Qiagen, Hilden, Germany), the cDNA synthesis kit (MBI Fermentas, St. Leon-Rot, Germany), RNA (1ug) was DNase treated at room temperature and reverse transcribed using superscript RTase and random primers, according to the manufacturer's protocol. mRNA expression was analyzed using Taqman primer/probe (Applied Biosystems) and multiplexed with 18S to account for total loading. Relative mRNA amounts were calculated using *CT* method[27] $\Delta CT = Ct$ target-*Ct* GAPDH, $\Delta\Delta CT = Ct$ treatment– Ct calibrator, where calibrator was the no-treatment group. *Ct* was then converted to fold change using the formula $2^{-\Delta\Delta CT}$. Quantitative PCR was performed using commercially obtained primers. Details of PCR primers are showed in table 1. (Table S1):

Table S1

Details of primers used in Real-time PCR

删除的内容: .

Gene name	Accession number	Forward primer	Reverse primer
Homo sapiens Vimentin	NM_003380	CTTCAGAGAGAGGAAGCCGA	ATTCCACTTTGCGTTCAAGG
Homo sapiens collagen, type I, alpha 1	NM_000088	CTTTGCATTCATCTCTCAAACTTAGTTTT	CCCCGCATGGGTCTTCA
Homo sapiens collagen, type IV, alpha 1	NM_001845.4	CTAATCACAAACTGAATGACTTGACTTCA	AAATGGCCCGAATGTGCTTA
Homo sapiens actin, alpha 2, smooth muscle, aorta (ACTA2)	NM_001613	CCGACCGAATGCAGAAGGA	ACAGAGTATTTGCGCTCCGAA
Homo sapiens aquaporin 5	NM_001651.3	TaqMan® probe no. Hs00387048_m1	
Homo sapiens surfactant protein C	NM_001172357.1	TaqMan® probe no.Hs00161628_m1	
Homo sapiens S100 calcium binding protein A4 (S100A4)	NM_002961.2	TaqMan® probe no.Hs00243202_m1	

Statistical Analysis

Data were normally distributed and analyzed by analysis of variance with Tukey's test for *post hoc* comparisons using Minitab 14.0 (Minitab, State College, PA). P value <0.05 was considered significant. Data are expressed as mean (SE).

Results

Figure S1. Effect of RvD1 upon effects of soluble Fas-ligand and TNF-alpha on proliferation and cell viability

A: sFasL and TNF-α inhibited cellular proliferation compared with control media–treated cells. This effect was attenuated by 100 nM RvD1. Experiments were performed using cells from 3 donors.

B: Cellular viability of AT II cells was reduced 24 hours after treatment with 100 ng/mL sFasL or (and) 100 ng/mL TNF-α. Pre-treatment with RvD1 at 100nM significantly increased the viability of sFasL or (and) TNF-α treated cells after 24 hours.

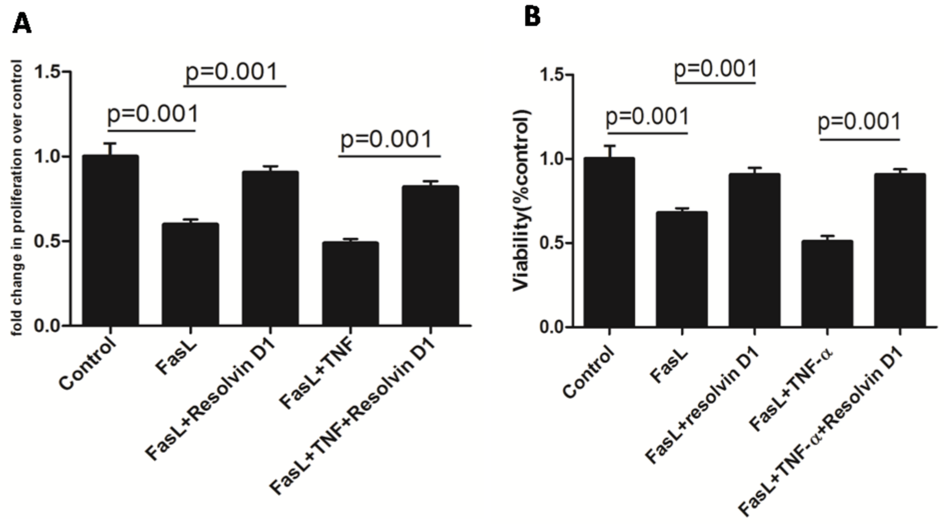
A
fold change in proliferation over control
1.5
1.0
0.5
0.0
p=0.001
p=0.001
p=0.001
Control
FasL
FasL+Resolvin D1
FasL+TNF
FasL+TNF+Resolvin D1
B
Viability(%control)
1.5
1.0
0.5
0.0
p=0.001
p=0.001
p=0.001
Control
FasL
FasL+resolvin D1
FasL+TNF-α
FasL+TNF-α+Resolvin D1
